# Correction: Bacchi et al. First Report on the Presence of Toxic Metals and Metalloids in East Asian Bullfrog (*Hoplobatrachus rugulosus*) Legs. *Foods* 2022, *11*, 3009

**DOI:** 10.3390/foods14183225

**Published:** 2025-09-17

**Authors:** Emanuela Bacchi, Gaetano Cammilleri, Marina Tortorici, Francesco Giuseppe Galluzzo, Licia Pantano, Vittorio Calabrese, Mariagrazia Brunone, Antonio Vella, Andrea Macaluso, Gianluigi Maria Lo Dico, Vincenzo Ferrantelli

**Affiliations:** 1 Istituto Zooprofilattico Sperimentale della Sicilia “A. Mirri”, Via Gino Marinuzzi 3, 90129 Palermo, Italy; 2 Dipartimento di Scienze Biomediche e Biotecnologiche, Università Degli Studi di Catania, Torre 11 Biologica Via Santa Sofia 97, 95123 Catania, Italy; calabres@unict.it; 3 Freelance Biologist, 90142 Palermo, Italy

## Adjustment of Authorship Order and Affiliation

In the original publication [1], the author list and the affiliation for Mariagrazia Brunone were published incorrectly. The correct author order and affiliations are as follows:

Emanuela Bacchi ^1^, Gaetano Cammilleri ^1,^*, Marina Tortorici ^1^, Francesco Giuseppe Galluzzo ^1^, Licia Pantano ^1^, Vittorio Calabrese ^2^, Mariagrazia Brunone ^3^, Antonio Vella ^1^, Andrea Macaluso ^1^, Gianluigi Maria Lo Dico ^1^ and Vincenzo Ferrantelli ^1^

^1^  Istituto Zooprofilattico Sperimentale della Sicilia “A. Mirri”, Via Gino Marinuzzi 3, 90129 Palermo, Italy
^2^  Dipartimento di Scienze Biomediche e Biotecnologiche, Università Degli Studi di Catania, Torre 11 Biologica Via Santa Sofia 97, 95123 Catania, Italy
^3^  Freelance Biologist, 90142 Palermo, Italy
* Correspondence: gaetano.cammilleri86@gmail.com

## Author Contributions

Project administration was conducted by Vincenzo Ferrantelli.

The authors state that the scientific conclusions are unaffected. This correction was approved by the Academic Editor. The original publication has also been updated.
